# The effects of parental psychological control and career decision-making self-efficacy on college students’ employment anxiety: the mediating role of motivational regulation

**DOI:** 10.3389/fpsyg.2026.1947477

**Published:** 2026-10-20

**Authors:** Pengfei Guo, Chun Wang, Hailong Fu, Jian Zhang

**Affiliations:** Student Affairs Office, Qingdao University, Qingdao, China

**Keywords:** career decision-making self-efficacy, employment anxiety, external regulation, identified regulation, parental psychological control, self-determination theory

## Abstract

Employment anxiety has become an important issue affecting college students’ psychological well-being and career development, yet its underlying mechanisms require further investigation. Based on Self-Determination Theory (SDT), this study examined associations of parental psychological control and career decision-making self-efficacy on college students’ employment anxiety, and further explored the mediating roles of identified regulation and external regulation. A questionnaire survey was conducted among 509 junior undergraduate students facing the upcoming graduation and job-search period. Participants completed the Future Employment Anxiety Scale, Parental Psychological Control Scale, Career Decision-Making Self-Efficacy Scale, and Work Extrinsic and Intrinsic Motivation Scale (WEIMS). Structural equation modeling and the Bootstrap method were employed to examine the relationships among the variables. The results showed that: (1) Parental psychological control was significantly and positively associated with college students’ employment anxiety, whereas career decision-making self-efficacy significantly and negatively associated with employment anxiety in statistical prediction; (2) Parental psychological control was significantly and negatively associated with identified regulation and external regulation, while career decision-making self-efficacy was significantly and positively associated with both types of motivational regulation; (3) Identified regulation and external regulation served as parallel statistical mediators in the associations between parental psychological control, career decision-making self-efficacy, and employment anxiety; and (4) Further analysis revealed that both types of motivational regulation showed masking patterns opposite to the directions of the direct statistical effects, suggesting that different forms of motivational regulation may be simultaneously linked to career engagement and higher anxiety in employment contexts. This study elucidates the psychological mechanisms through which family environment, individual psychological resources, and motivational regulation are jointly associated with employment anxiety, providing theoretical implications for employment-related psychological interventions in higher education.

## 1 Introduction

In recent years, intensifying employment competition, shifting economic conditions, and continuous adjustments in labor market demand have contributed to mounting employment pressure among college students (Huang et al., 2024; Qian et al., 2024; Yang, 2025; Zheng and Yan, 2024). Employment anxiety has consequently become a prevalent psychological challenge confronting university graduates (Yang et al., 2025; Peng et al., 2024). Employment anxiety not only impairs college students’ quality of career decision-making and enthusiasm for job hunting (Li and He, 2025; Qian et al., 2024), but also undermines their career adaptability, mental health, and long-term professional development (Bai et al., 2025; Zheng, 2024). As for its predictors, employment anxiety is shaped both by long-term family influences and moderated by individuals’ own psychological resources (Bozgeyikli et al., 2024; Wan et al., 2024; Pan et al., 2025). Accordingly, explaining college students’ employment anxiety from an integrated perspective of environmental and individual factors enables a more comprehensive understanding of its formation mechanism.

The family constitutes the primary socialization environment throughout individual development (Winnicott and Nussbaum, 2012; Garbarino, 2017) and exerts persistent, far-reaching impacts on career development (Whiston and Keller, 2004; Beauregard, 2007; Joseph, 2012). In recent years, compared with positive familial factors such as family support, parental psychological control has attracted growing scholarly attention (Williams and McKinney, 2024; Zheng and Chen, 2025). Parental psychological control refers to parents’ attempts to regulate children’s thoughts and behaviors through tactics including emotional withdrawal, guilt induction, and excessive interference. Fundamentally, it functions to constrain individuals’ autonomy (Barber, 1996; Soenens and Vansteenkiste, 2010). Conceptually a general parenting style construct, this study focuses on its manifestation in the career development domain.

Self-Determination Theory (SDT) posits that the needs for autonomy, competence, and relatedness represent basic psychological needs facilitating optimal human functioning (Deci and Ryan, 2008; Vansteenkiste and Ryan, 2013). When parents adopt psychologically controlling parenting, individuals’ need for autonomy is readily frustrated, eliciting adverse psychological outcomes such as anxiety and stress (Soenens and Vansteenkiste, 2010; Inguglia et al., 2022; Wei et al., 2021). Beyond family context, personal psychological resources serve as critical predictors of employment anxiety. Among these resources, career decision-making self-efficacy describes individuals’ confidence in completing career-related tasks, including career exploration, career planning, information seeking, and career choice (Gianakos, 2001). Social Cognitive Career Theory (SCCT) identifies self-efficacy as a core variable shaping career behaviors and development (Lent and Brown, 1996; Lent, 2005). Higher career decision-making self-efficacy strengthens individuals’ confidence in confronting vocational challenges (Xin et al., 2020), promotes career exploratory behaviors (Blustein, 1989; Pham et al., 2024), and reduces career decision-making difficulties as well as employment anxiety (Söner and Yılmaz, 2025).

Existing research has predominantly examined linear associations between career decision-making self-efficacy and employment anxiety (Müceldili et al., 2025; Dou et al., 2026). Nevertheless, simple unidirectional pathways fail to capture the complex psychological processes observed in reality. Within job-seeking contexts, individuals’ emotional reactions are not directly determined by external environments or internal capabilities (Lazarus and Folkman, 1984); instead, they are mediated by processes of motivational internalization (Turner et al., 2022). According to SDT, motivation evolves along an internalization continuum ranging from external control to autonomous functioning, encompassing external regulation, introjected regulation, identified regulation, integrated regulation, and intrinsic motivation (Deci and Ryan, 2000; Ryan and Deci, 2017). In employment settings, vocational behaviors are strongly goal-oriented and subject to practical constraints (Zhen et al., 2022), and motivation typically manifests in two fundamental forms: controlled motivation driven by external pressures and contingent outcomes, and autonomous motivation grounded in personal endorsement of vocational goals (Vansteenkiste et al., 2004, 2005). Prior studies have confirmed that parental psychological control predicts individuals’ behavioral regulation patterns (Wei et al., 2021). Motivational sources underlying vocational behaviors can be distinguished via different modes of motivational regulation (Guay, 2005), which are correlated with the degree of internalization of individuals’ career motivation (Jiang et al., 2022). Even so, few studies have systematically investigated how motivational regulation shapes emotional outcomes such as employment anxiety. Particularly for university graduates, job search may be motivated by the pursuit of financial independence (Zhao et al., 2023) or by value endorsement toward career advancement (Pshembayeva et al., 2022; Quinlan and Renninger, 2022). It follows that distinct motivational regulation patterns may alter individuals’ perceptions and psychological experiences surrounding job pursuit. Therefore, further inquiry into the mechanisms through which different types of motivational regulation operate in the development of employment anxiety is warranted.

Taken together, grounded in Self-Determination Theory (SDT), this study constructs a parallel mediation model with parental psychological control and career decision-making self-efficacy as independent variables, identified regulation and external regulation as parallel mediators. It aims to: (1) apply the SDT motivational framework to the context of college students’ employment anxiety; (2) examine the parallel mediating roles of two types of motivational regulation; (3) link the SCCT perspective of career self-efficacy with the SDT framework of motivational regulation. This study explores the statistical transmission mechanisms underlying employment anxiety and offers implications for university career mental health interventions.

## 2 Literature review

### 2.1 Parental psychological control and employment anxiety

A wealth of research has explored the association between parental psychological control and college students’ psychological adjustment. Schaefer (1965) first conceptualized parental psychological control, defining this parenting style as implicit manipulation of children through guilt induction, emotional withdrawal and authoritative interference, which essentially restricts individuals’ psychological autonomy. Cross-cultural research indicates that parental psychological control is more prevalent among Chinese parents compared with Western parents (Ng et al., 2014; Wuyts et al., 2015).

Parental psychological control is significantly and positively correlated with individuals’ anxiety, meaning that higher psychological control is generally accompanied by greater anxious experiences (Chyung et al., 2022; Inguglia et al., 2022; Lin and Guo, 2024). Theoretically, Self-Determination Theory (SDT) proposes that frustration of basic psychological needs, particularly the need for autonomy, is closely linked to negative emotional experiences and poor psychological adjustment (Deci and Ryan, 2000; Ryan and Deci, 2017). Psychological control hinders the satisfaction of individuals’ basic psychological needs including autonomy and competence (Wu et al., 2018). Further evidence reveals that psychological control disrupts behavioral planning and elevates anxiety by thwarting psychological need satisfaction (Wei et al., 2021; Liu et al., 2022). Studies situated within employment contexts also demonstrate that parental psychological control exerts adverse influences on college students’ interpersonal and social adjustment (Li, 2021). In addition, indigenous Chinese case studies confirm that parental psychological control and excessive parental career expectations significantly amplify university students’ career uncertainty and anxiety (Jiang and Song, 2023). Especially amid intensifying employment competition and mounting structural pressure, the emotional burden stemming from parental psychological control becomes more prominent, rendering it a vital familial factor shaping college students’ vocational psychology.

Based on the above theories and empirical findings, Hypothesis 1 (H1) is proposed: Parental psychological control is positively associated with college students’ employment anxiety.

### 2.2 Career decision-making self-efficacy and employment anxiety

Career decision-making self-efficacy was initially defined by Taylor and Betz (1983) as individuals’ beliefs or confidence in successfully completing all tasks required for career decision-making. According to Social Cognitive Career Theory, self-efficacy serves as a core cognitive variable governing vocational behaviors and career development; it shapes people’s behavioral choices, effort expenditure and persistence when confronting career-related tasks (Lent et al., 1994). Accordingly, individuals with higher career decision-making self-efficacy tend to cope with employment challenges with a positive mindset and exhibit stronger career adaptability (Alkal, 2025).

Consistent empirical evidence supports a stable direct negative predictive effect between the two variables. For instance, Li and He (2025) found in research on college students that career decision-making self-efficacy acts as a crucial internal predictor of employment anxiety. When individuals lack confidence in their capacity to make career decisions, they are more likely to experience a sense of helplessness and worry in a competitive job market, which further intensifies employment anxiety. Söner and Yılmaz (2025) verified this direct link among adolescents and populations undergoing career transitions: individuals with greater career decision-making self-efficacy possess more confidence to overcome various career obstacles and report substantially lower career anxiety; conversely, insufficient self-efficacy directly raises the risk of vocational anxiety.

Therefore, Hypothesis 2 (H2) is proposed: Career decision-making self-efficacy is negatively associated with college students’ employment anxiety.

### 2.3 Mechanisms of motivational regulation between parental psychological control, career decision-making self-efficacy and employment anxiety

College students’ job-seeking behaviors are not only shaped by vocational goals but also carry distinct practical functions. For undergraduates about to enter the labor market, extrinsic outcomes such as income, job stability and prospective career opportunities constitute vital considerations when engaging in employment-related activities. Meanwhile, individuals gradually develop an understanding of the value embedded in vocational goals and link job search to their personal developmental plans. Self-Determination Theory posits that behaviors vary in the degree of motivational internalization. External regulation describes behavior driven predominantly by extrinsic consequences and external demands, whereas identified regulation refers to individuals’ comprehension and voluntary acceptance of behavioral value (Deci and Ryan, 2000; Gagné and Deci, 2005). Furthermore, compared with more autonomous forms of motivation, controlled motivation is generally associated with higher negative affect, while personal endorsement of goal value facilitates adaptive psychological functioning (Ryan and Deci, 2017; Howard et al., 2021). Nevertheless, workplace characteristics moderate the associations between distinct motivation types and mental health outcomes (Gillet et al., 2016). Research within vocational domains similarly reveals divergent links between the quality of work motivation and individuals’ psychological well-being as well as negative emotions (Gagné and Deci, 2005; Vansteenkiste et al., 2005). Notably, when individuals strongly endorse a certain goal, the goal becomes closely tied to their personal values and self-concept, prompting them to devote substantial psychological resources to pursuing goal attainment (Sheldon and Elliot, 1999; Koestner et al., 2008). Amid fierce employment competition and uncertain future prospects, intense goal focus may further amplify anxiety regarding employment outcomes. Accordingly, within job-seeking contexts, identified regulation simultaneously reflects endorsement of goal value and heightened concern over outcomes, generating complex associations with employment anxiety. In short, external regulation and identified regulation represent two distinct modes of motivational regulation: outcome-oriented extrinsic motivation and internalization of vocational goal values. Distinguishing between them facilitates deeper understanding of the mechanisms through which parental psychological control and career decision-making self-efficacy are associated with employment anxiety.

#### 2.3.1 The mediating role of external regulation

External regulation denotes behavior driven by extrinsic rewards, social expectations, or the desire to avoid adverse consequences. It represents the least autonomous form of motivation proposed in Self-Determination Theory (Deci and Ryan, 2000). In employment contexts, college students may engage in job hunting for practical concerns including financial security, family expectations and social evaluation, making external regulation a crucial predictor of vocational psychology.

Both parental psychological control and career decision-making self-efficacy may shape levels of external regulation. First, characterized by control, pressure and external evaluation, parental psychological control frustrates individuals’ need for autonomy and hinders the internalization of behavioral goals, predisposing individuals to regulate conduct according to external standards. Studies focusing on Chinese university students confirm that parental psychological control positively predicts frustration of basic psychological needs, reshaping individuals’ behavioral regulation patterns and orienting them toward controlled motivation (Wei et al., 2021). In job-seeking scenarios, students exposed to higher parental psychological control struggle to internalize job search as a personal developmental objective. Instead, they tend to perceive job hunting as a task meant to satisfy parental expectations and evade external pressure, manifesting higher levels of external regulation.

Second, as a key psychological resource during career development, career decision-making self-efficacy also predicts modes of motivational regulation. Drawing on Career Construction Theory, Jiang et al. (2022) demonstrated that career decision-making self-efficacy correlates with the internalization of vocational motivation. Individuals with high self-efficacy are more likely to develop autonomous career motivation, whereas those with low self-efficacy tend to exhibit controlled motivational tendencies. Therefore, career decision-making self-efficacy shapes external regulation by influencing autonomous experiences in vocational behaviors. Research on basic psychological need satisfaction further indicates that enhanced feelings of competence and autonomy constitute the foundation of motivational internalization, and career decision-making self-efficacy embodies competence within the vocational domain (Xu et al., 2025).

In terms of outcomes, external regulation serves as a critical psychological predictor of employment anxiety. Job preparation is marked by pervasive uncertainty and competitive pressure. When job-seeking behavior is primarily propelled by external requirements, others’ expectations or extrinsic evaluation, vocational goals lack stable intrinsic value support. Individuals are prone to frame job pursuit as a source of stress, which intensifies anxious feelings. For instance, prior studies have found that controlled motivation correlates with maladaptive vocational outcomes including career indecision and poor career adjustment (Paixão and Gamboa, 2022). Consistent evidence exists in other achievement-related stressful settings: low-autonomy motivation shapes how individuals perceive tasks and subsequently elevates anxiety (Li and Ding, 2024). From the perspective of Control-Value Theory (Pekrun, 2006), individuals under external regulation experience low agency over job-seeking behavior and derive value from extrinsic sources. They are more likely to appraise employment pressure as threatening, which ultimately exacerbates anxiety.

To sum up, external regulation may statistically mediate the associations of parental psychological control and career decision-making self-efficacy with employment anxiety. That is, external regulation mediates the associations between the two antecedent variables and employment anxiety. Accordingly, the following hypothesis 3 (H3) is proposed: External regulation statistically mediates the respective associations of parental psychological control and career decision-making self-efficacy with college students’ employment anxiety.

#### 2.3.2 The mediating role of identified regulation

Identified regulation represents a relatively autonomous form of extrinsic motivation within Self-Determination Theory. It refers to the process in which individuals recognize the intrinsic value of a behavior for personal development, voluntarily embrace its goals, and integrate those goals into their own value systems (Deci and Ryan, 2000; Ryan and Deci, 2017). Unlike controlled motivation driven by external pressures or social expectations, identified regulation reflects genuine comprehension and acceptance of goal meaning, serving as a vital psychological mechanism facilitating the internalization of vocational goals and proactive career engagement. Gagné and Deci (2005) argued that individuals are more likely to engage in activities autonomously when they can transform external demands into personally endorsed value objectives. Drawing on Self-Determination Theory, Guay et al. (2003) found that the quality of motivation in career choices is closely linked to career exploration and career indecision; higher autonomous motivation fosters more adaptive career development trajectories. In the context of university graduate job hunting, identified regulation captures the extent to which students view career preparation and vocational growth as personally meaningful pursuits, which in turn shapes their psychological adjustment amid employment stress.

Family environments constitute a key social determinant of motivational internalization. The formation of autonomous goal identification largely hinges on whether one’s basic need for autonomy is satisfied in their surroundings. Compared with autonomy-supportive parenting, psychologically controlling family contexts undermine autonomous experiences and hinder the genuine internalization of behavioral values (Soenens and Vansteenkiste, 2010). In a 2025 meta-analysis covering 238 studies grounded in the dual-process model of Self-Determination Theory, Bradshaw et al. (2025) confirmed that parental psychological control robustly predicts poor developmental outcomes, whereas autonomy support correlates with positive functioning. This finding solidifies the stable adverse impact of psychological control as a contextual barrier to autonomous growth. Accordingly, university students raised in psychologically controlling households tend to make career decisions based on familial expectations and external standards, rather than cultivating self-endorsed vocational identities, which suppresses their level of identified regulation.

Beyond family context, career decision-making self-efficacy acts as a critical individual factor shaping the internalization of career motivation. While few studies have directly examined the link between career decision-making self-efficacy and identified regulation, abundant evidence indicates significant positive correlations between self-efficacy and vocational identity (Choi et al., 2012; Middleton, 2017; Chang et al., 2025). Paixão and Gamboa (2022) further verified that autonomous motivation centered on identified regulation alleviates career indecision. Combined with the established logic that self-efficacy fuels career exploration, it can be inferred that stronger career decision-making self-efficacy deepens individuals’ recognition of vocational values and shifts their motivational patterns toward more autonomous identified regulation.

Furthermore, autonomous motivation such as identified regulation shapes affective experiences. Cross-domain research has consistently demonstrated that higher levels of autonomous motivation correspond to fewer negative emotions, a pattern replicated across academic and foreign language learning settings (Wang and Liu, 2022).

In summary, identified regulation may function as a core psychological pathway linking parental psychological control and career decision-making self-efficacy to employment anxiety. On one hand, parental psychological control impedes the internalization of vocational goals by eroding autonomous perceptions, thereby elevating anxiety risk. On the other hand, robust career decision-making self-efficacy strengthens one’s sense of agency over career development and promotes the formation of intrinsic vocational values. Nevertheless, contextual workplace characteristics moderate the associations between distinct motivation types and mental health outcomes (Gillet et al., 2016). High identified regulation may amplify dedication to career objectives and alter psychological responses to job hunting. Yet the directional nature of this relationship remains underexplored within the highly outcome-oriented employment context. Thus, Hypothesis 4 (H4) is proposed: Identified regulation mediates the respective relationships of parental psychological control and career decision-making self-efficacy with college students’ employment anxiety.

### 2.4 Summary of research and theoretical model

#### 2.4.1 Summary of existing research

Taken together, abundant empirical findings have been accumulated regarding the predictors of college students’ employment anxiety; nevertheless, three research gaps remain to be addressed.

Before outlining the gaps, we clarify the rationale for modeling parental psychological control and career decision-making self-efficacy as parallel independent predictors. Parental psychological control represents a distal developmental contextual factor, while career decision-making self-efficacy is a proximal individual psychological resource. The core focus of this study is to examine their distinct mediating pathways through motivational regulation, rather than to test a sequential causal chain from family context to self-efficacy.

Within this framework, Self-Determination Theory (SDT) serves as the overarching theoretical perspective for explaining the relationships among social-contextual factors, motivational regulation, and employment anxiety. Social Cognitive Career Theory (SCCT) complements SDT by specifying the role of career decision-making self-efficacy as an individual cognitive resource in the career domain. Thus, SDT provides the primary explanation for the motivational mechanism, whereas SCCT helps contextualize the role of career-related self-efficacy. Together, these perspectives provide a coherent framework for examining how parental psychological control and career decision-making self-efficacy are statistically associated with employment anxiety through motivational regulation.

First, insufficient research integrates predictors from dual perspectives. Prior studies have separately verified that parental psychological control positively predicts anxious emotions (Chyung et al., 2022; Jiang and Song, 2023), while career decision-making self-efficacy exerts a protective negative effect on employment anxiety (Li and He, 2025; Söner and Yılmaz, 2025). However, these two sets of variables are mostly examined in isolation and rarely incorporated into a unified analytical framework. Consequently, the joint mechanisms through which environmental and individual factors shape employment anxiety cannot be comprehensively uncovered. Second, the mediating mechanisms of motivational internalization remain underexplored. From the perspective of Self-Determination Theory, motivational internalization serves as a core psychological pathway linking external environments, individual cognition, and emotional outcomes (Deci and Ryan, 2000). Existing evidence indicates that parental psychological control impedes the process of motivational internalization (Wei et al., 2021), and career decision-making self-efficacy is correlated with the internalization of career motivation (Jiang et al., 2022). Even so, few studies have systematically examined how these two variables predict employment anxiety via motivation at distinct internalization levels. Third, limitations exist in research outcomes and model construction. Most existing studies on vocational motivation focus on behavioral outcomes such as career exploration and career indecision (Paixão and Gamboa, 2022), with limited attention paid to emotional outcomes represented by employment anxiety. Moreover, relevant research generally tests the effects of a single motivational type, lacking simultaneous comparisons of the mediating roles of identified regulation and external regulation. The complex transmission mechanisms embedded within job-seeking contexts are thus insufficiently understood.

#### 2.4.2 Theoretical model

See Figure 1.

**Figure 1:**
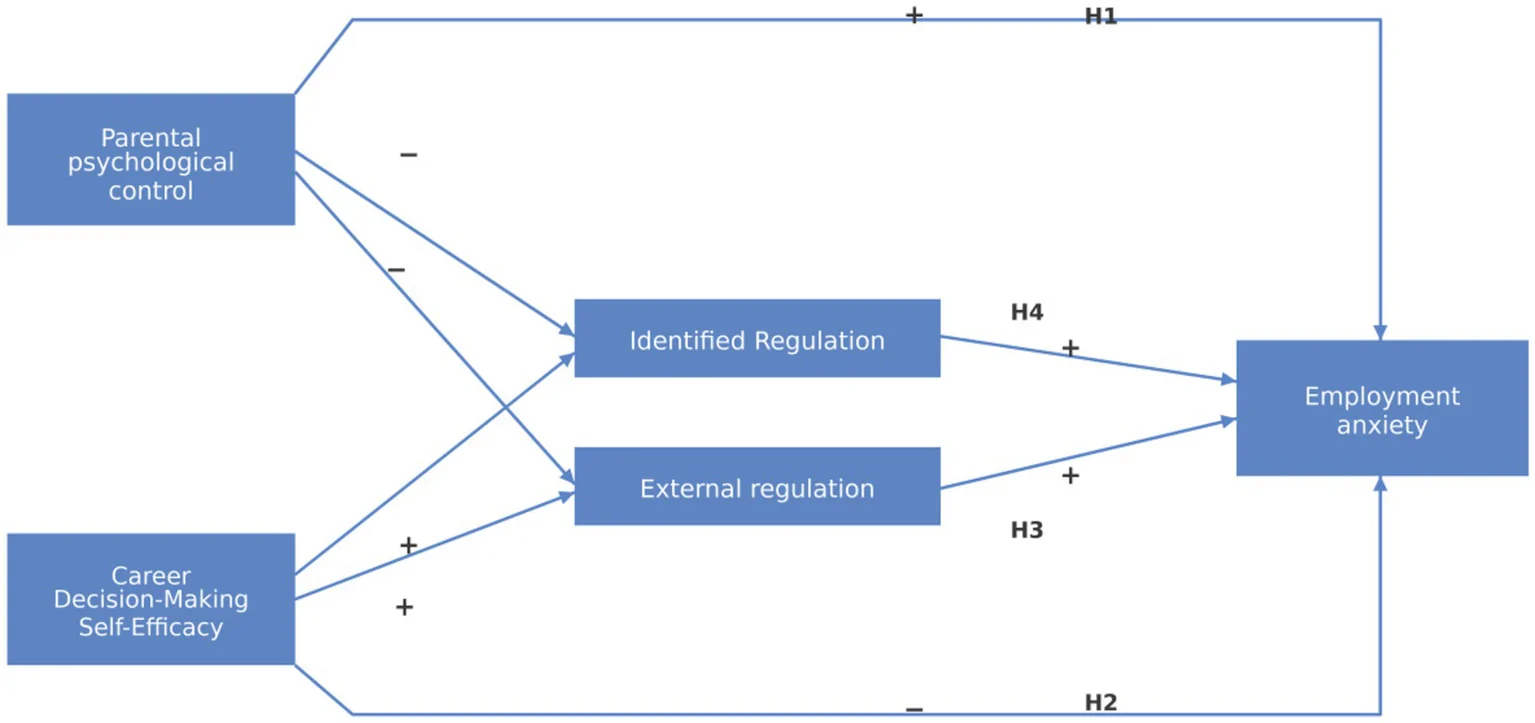
Theoretical research model.

## 3 Method

### 3.1 Participants

Participants in this study were junior students from a university in China who were about to enter the graduation job-hunting season. A convenience sampling method was adopted to distribute online questionnaires via the Wenjuanxing platform. A total of 885 raw questionnaires were collected, and two-step data cleaning was implemented sequentially. First, invalid questionnaires with careless responses featuring overly short completion time were excluded. Second, a Z-score method was adopted for outlier detection, removing extreme samples with absolute standardized scores |Z|>3. Ultimately, 509 valid questionnaires were obtained, yielding an effective response rate of 57.51%.

The demographic characteristics of the sample were as follows: in terms of gender, there were 199 males (39.1%) and 310 females (60.9%). Majors covered science and engineering, humanities and social sciences, and medicine. This study strictly complied with ethical guidelines for psychological research. All participants took part voluntarily. The questionnaires were fully anonymous, and an informed consent statement was presented at the beginning to clarify that data were used solely for academic research and would be kept strictly confidential.

### 3.2 Measures

Because the instruments were originally developed in English, their items were translated into Chinese before administration. The translation was conducted with attention to semantic equivalence and the appropriateness of wording for Chinese university students. For instruments that were contextually adapted, such as the Parental Psychological Control Scale and the Future Employment Anxiety Scale, the Chinese items were further revised to fit the specific career and employment context while retaining the core meaning of the original constructs. The psychometric properties of the resulting measures were subsequently examined using reliability analysis and confirmatory factor analysis. All scales in the present study adopted a 5-point Likert scoring format, ranging from 1 (strongly disagree) to 5 (strongly agree). Higher scores on each scale indicated higher levels of the corresponding variable. Note that a formal back-translation procedure was not performed in the present study.

#### 3.2.1 Future employment anxiety scale

The Future Employment Anxiety Scale (FEAS), developed by Wang et al. (2025), was utilized. Consisting of four dimensions with 17 items, the scale assesses individuals’ anxious emotions, worry, and psychological pressure arising from anticipated employment scenarios. Minor revisions were made to certain statements to fit the employment context of university students in China.

The original scale contained 17 items (original item numbers 7–22). Based on item analysis (critical ratio CR<3) and preliminary confirmatory factor analysis (factor loading <0.5, modification index MI>3.84), several items exhibited high redundancy, mainly reflecting uncertainty and worry about future employment outcomes with limited unique information contribution. Considering that high content overlap among items may lead to correlated error terms, Items 14, 17, 20 and 22 were removed successively. Finally, 13 items were retained for formal data analysis.

#### 3.2.2 Work extrinsic and intrinsic motivation scale

The Work Extrinsic and Intrinsic Motivation Scale (WEIMS) was adopted (Tremblay et al., 2009). The original scale includes five dimensions of motivational regulation. Given that this study focused on the formation of vocational goals and behavioral driving mechanisms within job-seeking contexts, only external regulation and identified regulation were examined. Job preparation among university students constitutes an essential stage of future career development; its goal formation and behavioral regulation share substantial consistency with vocational motivation. Therefore, WEIMS was used to measure motivational regulation in the career development context without modifications to item wording.

Each dimension contained 3 items, resulting in 6 items in total. The identified regulation subscale measured autonomous motivation stemming from personal recognition of vocational value and developmental significance. The external regulation subscale measured motivation driven by external rewards and punishments, material benefits, and evaluations from others.

#### 3.2.3 Career decision-making self-efficacy scale

The Career Decision-Making Self-Efficacy Scale (CDMSE) was employed to measure individuals’ subjective confidence in their ability to complete career decision-making tasks, including information collection, goal selection, plan formulation, and problem solving. It is a classic and well-established instrument in vocational psychology research. No adjustments were made to the original items.

The initial scale contained 25 items. After preliminary screening, one item was eliminated, leaving 24 items. Subsequent confirmatory factor analysis revealed unsatisfactory model fit indices including χ^2^, CFI and TLI. Items 50 and 52 were further deleted, and 22 items were retained for formal analysis.

#### 3.2.4 Parental psychological control scale

The Parental Psychological Control Scale, originally developed by Barber (1996), was utilized to assess parental psychological control. The original scale is not specific to career contexts. Minor contextual revisions were performed in the present study to adapt the scale to college students’ career development scenarios, while the core meaning of the original items was retained. The original scale included 8 items. Item analysis indicated considerable content redundancy among some items. To improve the scale’s construct validity, Items 63 and 64 were removed, and 6 items were retained for formal analysis.

### 3.3 Procedure

Standardized online data collection was conducted via the Wenjuanxing platform following the procedure below.

Unified instructions and informed consent information were displayed at the start of each questionnaire, clarifying research objectives, anonymity and confidentiality principles, voluntary participation, and participants’ right to terminate the survey at any time. Questionnaires were presented in a fixed order: demographic information, the Future Employment Anxiety Scale, the Work Extrinsic and Intrinsic Motivation Scale, the Career Decision-Making Self-Efficacy Scale, and the Parental Psychological Control Scale. After questionnaire collection, independent researchers completed data cleaning and invalid sample exclusion. The final analytical dataset was generated only after cross-verification produced consistent results.

### 3.4 Data processing and analysis

SPSS 26.0 and Amos 24.0 statistical software were jointly adopted to conduct all data analyses.

SPSS 26.0 was used for data preprocessing and basic statistical analyses, including screening by response duration, outlier elimination via Z-score, descriptive statistics, reliability test using Cronbach’s α coefficient, exploratory factor analysis (KMO test and Bartlett’s test of sphericity), and Pearson correlation analysis. Harman’s single-factor test was performed to examine and control common method bias.

Amos 24.0 was utilized to establish a parallel mediation structural equation model. Parental psychological control and career decision-making self-efficacy were set as two independent variables; identified regulation and external regulation served as parallel mediators; employment anxiety was treated as the dependent variable. The bias-corrected Bootstrap method with 5,000 resamples was adopted to test the total indirect effect and each specific mediating path effect, and masking effects were further examined.

The bias-corrected percentile Bootstrap method (5,000 resamples) was used to test the significance of mediating effects. Model fit was evaluated using widely accepted indices: χ^2^/df (good fit < 3; acceptable fit < 5), root mean square error of approximation (RMSEA; acceptable fit < 0.08; good fit < 0.05), comparative fit index (CFI; acceptable fit > 0.90; good fit > 0.95), Tucker-Lewis index (TLI; acceptable fit > 0.90), and standardized root mean square residual (SRMR; acceptable fit < 0.08).

## 4 Results

### 4.1 Common method bias test

All data in this study were collected via participants’ self-reports. Anonymous questionnaires and other procedural controls were implemented during data collection to mitigate common method bias. Statistically, Harman’s single-factor test was conducted after data collection.

An unrotated exploratory factor analysis was performed on all formal items. Nine common factors with eigenvalues greater than 1 were extracted. The first factor accounted for 28.80% of the total variance, below the critical threshold of 40%, indicating that common method bias exerted an acceptable influence on the present findings.

### 4.2 Descriptive statistics and gender differences

Independent-samples t-tests were conducted to examine gender differences across all variables. Results showed a highly significant gender difference for employment anxiety (t = −3.95, *p* < 0.001), with female students reporting significantly higher employment anxiety than male students. A significant gender difference was found for career decision-making self-efficacy (t = 2.36, *p* = 0.019); male students exhibited significantly higher career decision-making self-efficacy than female students. No significant gender differences emerged for identified regulation, external regulation, or parental psychological control (all ps > 0.05). Descriptive statistics for all core variables are presented in Table 1.

**Table 1:** Descriptive statistics of core variables (*n* = 509).

Variable	Mean (M)	Standard deviation (sd)	Skewness	Kurtosis
Employment anxiety	3.37	0.74	−0.27	0.69
Parental psychological control	2.18	0.91	0.58	−0.20
Identified regulation	3.96	0.66	−0.32	0.36
External regulation	4.27	0.53	−0.36	−0.39
Career decision-making self-efficacy	3.54	0.57	0.52	0.64

### 4.3 Reliability and validity tests

#### 4.3.1 Internal consistency reliability

Cronbach’s α coefficients were calculated to evaluate the internal consistency of each scale, as displayed in Table 2.

**Table 2:** Reliability results of all scales and subscales.

Scale / subscale	Final number of items	Cronbach’s α
Employment anxiety (after item deletion)	13	0.905
Parental psychological control (after item deletion)	6	0.918
Identified regulation	3	0.844
External regulation	3	0.671
Career decision-making self-efficacy (after item deletion)	22	0.953

The Cronbach’s α coefficients of employment anxiety, career decision-making self-efficacy, parental psychological control and identified regulation were all above 0.8, indicating satisfactory internal consistency. The α coefficient of external regulation was 0.671. Although this value was slightly lower than the conventional cutoff of 0.70, this subscale only contained three items. The composite reliability (CR) calculated from standardized factor loadings in confirmatory factor analysis reached 0.769, meeting the acceptable criterion of CR ≥ 0.6 proposed by Hair et al. (2010). Therefore, the internal consistency of this subscale was regarded as acceptable.

#### 4.3.2 Confirmatory factor analysis (construct validity)

AMOS 24.0 was used to perform confirmatory factor analysis on the baseline five-factor measurement model. The model fit indices were as follows: χ^2^/df = 4.082, below the acceptable threshold of 5; RMSEA = 0.078, less than 0.08; incremental fit indices CFI = 0.918 and TLI = 0.903, both exceeding 0.90; additionally, NFI = 0.895, GFI = 0.891, RMR = 0.065. Collectively, the five-factor measurement model achieved adequate fit, demonstrating favorable construct validity for the research variables.

### 4.4 Correlation analysis

Pearson correlation coefficients among core variables are presented in Table 3, with key findings summarized as follows. Parental psychological control was significantly and positively correlated with employment anxiety (*r* = 0.302, *p* < 0.001). Career decision-making self-efficacy exhibited a significant negative correlation with employment anxiety (*r* = −0.379, *p* < 0.001) and a significant negative correlation with parental psychological control (*r* = −0.150, *p* < 0.001), offering preliminary evidence for subsequent path analysis. Zero-order correlations between identified regulation, external regulation and employment anxiety were non-significant (*p* > 0.05). Both identified regulation and external regulation were significantly positively correlated with career decision-making self-efficacy and significantly negatively correlated with parental psychological control (all *p* < 0.05).

**Table 3:** Correlation matrix of core variables (*n* = 509).

Variable	1	2	3	4	5
1. Parental psychological control	1				
2. Employment anxiety	0.302***	1			
3. Identified regulation	−0.168**	0.037	1		
4. External regulation	−0.112*	0.077	0.583***	1	
5. Career decision-making self-efficacy	−0.150***	−0.379***	0.424***	0.297***	1

*p <* 0.05, ** *p* < 0.01, *** *p* < 0.001.

Zero-order correlations of identified regulation and external regulation with employment anxiety were non-significant (*p* > 0.05). This pattern will be further interpreted in combination with structural equation modeling results, as bivariate associations may be obscured when multiple predictors exert opposing influences. The presence of suppression effects will be formally tested based on the signs of direct and indirect effects in the SEM results.

Notably, the zero-order correlation between parental psychological control and career decision-making self-efficacy was relatively small in magnitude (*r* = −0.150, *p* < 0.001), indicating that the two are largely distinct constructs and supporting our decision to treat them as parallel independent predictors.

### 4.5 Test of main effects: hierarchical multiple regression analysis

Hierarchical regression analysis was conducted to examine the independent predictive effects of parental psychological control and career decision-making self-efficacy on employment anxiety. In Step 1, demographic control variables including gender and major category were entered (all valid participants were third-year undergraduates, and no between-group differences existed in grade, so grade was not included in the regression model). In Step 2, the independent variable parental psychological control was added. In Step 3, career decision-making self-efficacy was incorporated.

Collinearity diagnostics indicated that the variance inflation factor (VIF) values of all predictors ranged from 1.005 to 1.037, far below the cutoff value of 5, suggesting no serious multicollinearity. The Durbin-Watson statistic was 2.017, satisfying the assumption of independent residuals. Standardized residuals ranged from −3.613 to 3.450 and approximately followed a normal distribution. All prerequisites for regression analysis were satisfied. The results of hierarchical regression are presented in Table 4.

**Table 4:** Hierarchical regression results predicting employment anxiety (*N* = 509).

Predictor variable	Model 1 β	Model 2 β	Model 3 β
Control variable: Gender	0.181***	0.166***	0.132***
Control variable: Major category	0.109*	0.088*	0.069
Parental psychological control	—	0.289***	0.243***
Career decision-making self-efficacy	—	—	−0.324***
R^2^	0.042	0.124	0.225
Adjusted R^2^	0.038	0.119	0.219
ΔR^2^	0.042***	0.083***	0.101***

—— indicates the variable was not entered in this model; *p < 0.05, ***p < 0.001.

Model 1 only contained gender and major category as control variables, explaining 4.2% of the variance in employment anxiety. After adding parental psychological control in Model 2, the explanatory power increased significantly, accounting for an additional 8.3% of variance. Further inclusion of career decision-making self-efficacy in Model 3 yielded a total explained variance of 22.5%. The full model showed that parental psychological control was significantly and positively associated with employment anxiety, whereas career decision-making self-efficacy was significantly and negatively associated with employment anxiety. The predictive effect of major category on employment anxiety became non-significant.

Furthermore, both variables explained unique significant variance in employment anxiety after mutually controlling for one another, further reinforcing their roles as parallel predictors.

### 4.6 Test of parallel mediating effects

A parallel mediation structural equation model with two independent variables was established. Parental psychological control and career decision-making self-efficacy served as independent variables; identified regulation and external regulation acted as two parallel mediators; employment anxiety was the dependent variable. The overall model fit indices were as follows: χ^2^/df = 4.082, RMSEA = 0.078, CFI = 0.918, TLI = 0.903. All indices reached acceptable thresholds, indicating adequate model fit. The bias-corrected Bootstrap method with 5,000 resamples was adopted to test the significance of indirect effects.

#### 4.6.1 Test of direct path coefficients

Unstandardized regression coefficients, standard errors and significance results for each path are displayed in Table 5.

**Table 5:** Significance test of direct path coefficients in the model.

Path	Unstandardized estimate	Standard error	CR	*p*
Parental psychological control → Identified regulation	−0.075	0.028	−2.732	0.006
Parental psychological control → External regulation	−0.034	0.015	−2.263	0.024
Career decision-making self-efficacy → Identified regulation	0.473	0.055	8.532	<0.001
Career decision-making self-efficacy → External regulation	0.083	0.028	2.996	0.003
Identified regulation → Employment anxiety	0.273	0.058	4.676	<0.001
External regulation → Employment anxiety	0.456	0.118	3.878	<0.001
Parental psychological control → Employment anxiety (direct effect)	0.195	0.030	6.465	<0.001
Career decision-making self-efficacy → Employment anxiety (direct effect)	−0.581	0.067	−8.606	<0.001

The results indicated that parental psychological control was significantly and negatively associated with identified regulation and external regulation. Career decision-making self-efficacy was significantly and positively associated with both motivational mediators. Both identified regulation and external regulation were significantly and positively associated with employment anxiety. The direct statistical associations of the two independent variables with employment anxiety were significant.

#### 4.6.2 Bootstrap test of indirect effects

The bias-corrected Bootstrap method with 5,000 resamples was used to test the parallel mediating effects. Unstandardized indirect effects, standard errors and 95% bias-corrected confidence intervals for the four separate mediating paths are presented in Table 6. None of the confidence intervals contained zero, demonstrating that all four independent mediating effects were statistically significant.

**Table 6:** Bootstrap tests for four independent parallel mediating paths (*N* = 509), 5,000 resamples).

Mediating path	Unstandardized indirect effect	Standard error	95% Lower CI	95% Upper CI	Significance
Parental psychological control → Identified regulation → Employment anxiety	−0.0205	0.009	−0.041	−0.005	Significant
Parental psychological control → External regulation → Employment anxiety	−0.0155	0.008	−0.033	−0.002	Significant
Career decision-making self-efficacy → Identified regulation → Employment anxiety	0.1291	0.035	0.068	0.203	Significant
Career decision-making self-efficacy → External regulation → Employment anxiety	0.0378	0.016	0.011	0.073	Significant

Indirect effects were calculated as the product of the unstandardized path coefficient from the independent variable to the mediator and the coefficient from the mediator to the dependent variable. Identified regulation and external regulation served as parallel independent mediators, separately transmitting the statistical associations of the two independent variables with employment anxiety.

Classified by the sign of effects, the two indirect effects of parental psychological control via identified regulation and external regulation were negative. By contrast, the two indirect effects of career decision-making self-efficacy through the two types of motivational regulation were positive. Comparing the absolute magnitudes of the four paths, the indirect effect of the pathway career decision-making self-efficacy to identified regulation to employment anxiety was the largest, making it the most influential mediating channel in the model.

As illustrated in the table, the sign of each indirect mediating statistical effect was opposite to the sign of the corresponding direct effect, which constitutes the essential prerequisite for the emergence of suppression patterns. The total effects, direct effects, total indirect effects and the proportion of suppression offset for each independent variable are summarized in Table 7. Obvious suppression effects existed for both predictors. The positive direct statistical association of parental psychological control with employment anxiety was countervailed negatively via the two motivational mediators. Meanwhile, the negative direct statistical association of career decision-making self-efficacy with employment anxiety was offset positively by the mediating pathways, weakening the overall statistical predictive power of the independent variable toward employment anxiety. The 95% Bootstrap confidence intervals for the total effects of both independent variables excluded zero, indicating that the overall predictive effects of the predictors on employment anxiety remained statistically significant.

**Table 7:** Summary of total effects, direct effects, total indirect effects and suppression proportions of independent variables.

Predictor variable	Total effect	Direct effect	Total indirect effect	Suppression proportion	95% CI for total effect
Parental psychological control	0.159	0.195	−0.036	18.45%	[0.089, 0.237]
Career decision-making self-efficacy	−0.414	−0.581	0.167	28.74%	[−0.551, −0.285]

A comparison of the signs of direct effects and total indirect effects revealed that indirect effects had opposite signs to direct effects for both independent variables, satisfying the criteria for suppression effects. Following the statistical conventions for suppression effect analysis, the suppression proportion was calculated as the absolute value of the indirect effect divided by the absolute value of the direct effect, rather than the ratio of the indirect effect to the total effect.

The suppression proportion of parental psychological control was 18.45%, indicating that its positive direct predictive effect on employment anxiety was attenuated by 18.45% through the opposing motivational mediating pathways. The suppression proportion of career decision-making self-efficacy reached 28.74%, meaning its direct anxiety-alleviating negative effect was offset by 28.74% via the motivational mediators. The 95% confidence intervals for the total effects of both predictors excluded zero, which demonstrated that the overall predictive effects of the independent variables on employment anxiety remained statistically significant.

## 5 Discussion

### 5.1 Effects of parental psychological control and career decision-making self-efficacy on employment anxiety

This study first explored the associations of parental psychological control and career decision-making self-efficacy with college students’ employment anxiety. The results revealed that parental psychological control was significantly and positively associated with employment anxiety, whereas career decision-making self-efficacy was significantly and negatively associated with employment anxiety. These findings support previous arguments that family contextual factors and individual psychological resources are linked to vocational psychological adjustment.

Specifically, the positive statistical association between parental psychological control and employment anxiety aligns with Self-Determination Theory’s propositions regarding controlling social environments. Both early and recent studies have documented that psychologically controlling family contexts undermine individuals’ sense of autonomy and prevent them from making choices consistent with personal values, thereby elevating adverse psychological outcomes (Soenens and Vansteenkiste, 2010; Bradshaw et al., 2025). During the transitional period prior to graduation, career selection and planning constitute core developmental tasks for autonomy. When parents interfere excessively or exert emotional control over their children’s career decisions, students struggle to build autonomy and agency over vocational development. They are more likely to perceive job hunting as an external stressor, which in turn is linked to higher employment anxiety.

Meanwhile, the negative statistical association of career decision-making self-efficacy with employment anxiety confirms its function as a protective factor in vocational psychology. Existing evidence indicates that career decision-making self-efficacy is associated with stronger confidence in career exploration, information gathering, and career choice tasks, facilitating adaptive career development (Li and He, 2025; Söner and Yılmaz, 2025). The present study further demonstrates that amid high employment uncertainty, college students with higher career decision-making self-efficacy are more likely to maintain a sense of agency over their vocational trajectories. They tend to interpret employment challenges as solvable problems rather than uncontrollable threats, and consequently report lower employment anxiety.

### 5.2 The mediating roles of motivational regulation between parental psychological control, career decision-making self-efficacy and employment anxiety

This study further confirmed that external regulation and identified regulation exert significant statistical effects between the two predictors and employment anxiety, suggesting that motivational internalization acts as a critical psychological pathway linking external environments, personal psychological resources, and employment anxiety. This finding extends prior research, which has mainly focused on how vocational motivation shapes career exploration and decision-making behaviors, by expanding the mechanism of motivational regulation to the emotional outcome of employment anxiety.

First, parental psychological control significantly and negatively associated with both external regulation and identified regulation, indicating that controlling family environments hinder the development of college students’ vocational motivation. This result is consistent with Self-Determination Theory’s account of motivational internalization. Prior research suggests that whether individuals can transform external demands into personally endorsed goals largely depends on whether their basic need for autonomy is satisfied within social surroundings (Deci and Ryan, 2000; Soenens and Vansteenkiste, 2010). College students raised in psychologically controlling households tend to make career choices subject to external influences, which impedes the formation of stable vocational value identification and may also reduce their focus on extrinsic outcomes of job-seeking activities.

Second, career decision-making self-efficacy significantly and positively associated with external regulation and identified regulation, demonstrating that beliefs in one’s own career decision-making competence are linked to vocational goal formation and motivational development. Although few studies have directly examined the association between career decision-making self-efficacy and identified regulation, existing research has established close links between career decision-making self-efficacy, vocational identity, and career exploration (Choi et al., 2012; Chang et al., 2025). Accordingly, college students with higher career decision-making self-efficacy are more capable of recognizing the meaning of career development and establishing clearer vocational goals.

### 5.3 Further interpretation of the relationships between motivational regulation and employment anxiety

Notably, the present study found that both identified regulation and external regulation were significantly and positively associated with employment anxiety after controlling for antecedent variables. This finding differs from results of some Self-Determination Theory research, which generally suggests that autonomous motivation facilitates adaptive psychological functioning. A plausible explanation lies in the strong future outcome orientation and tangible pressure embedded within job-seeking contexts.

In terms of identified regulation, individuals integrate vocational goals into their personal value systems and invest substantial psychological resources in such goals. Prior studies have demonstrated that identified regulation promotes career exploration and alleviates career indecision (Guay et al., 2003; Paixão and Gamboa, 2022). Nevertheless, the current study reveals that strong endorsement of vocational goals may also entail heightened concern over career outcomes. Under conditions of uncertainty regarding goal attainment, intense identification with vocational objectives is associated with amplified focus on employment results. When employment prospects remain unpredictable, such concern may translate into psychological stress and elevated employment anxiety.

As for external regulation, the construct measured in this study reflects an outcome orientation centered on financial rewards and livelihood security obtained via employment. When college students regard employment as a primary route to material gains and stable living conditions, their job-seeking behaviors hinge heavily on the realization of future outcomes. Amid fierce competition in the labor market, preoccupation with remuneration and job stability is associated with higher sensitivity to employment results, rendering individuals more susceptible to anxiety.

Accordingly, this study indicates that the association between motivational regulation and employment anxiety cannot be simplified into the pattern that autonomous motivation reduces anxiety while controlled motivation increases anxiety; instead, it should be interpreted within specific contextual settings. During this highly outcome-oriented transitional phase before graduation, investment in vocational goals and focus on extrinsic outcomes may simultaneously foster proactive behavioral tendencies and generate psychological strain. Importantly, this conditional association observed in the present model does not contradict core tenets of Self-Determination Theory.

### 5.4 Theoretical interpretation of suppression effects

Further analyses confirmed suppression patterns for both forms of motivational regulation, meaning indirect statistical operated in the opposite direction of corresponding direct effects. The two suppression pathways may reflect distinct psychological processes. Parental psychological control may be linked to lower anxiety by weakening individuals’ investment in vocational goals, whereas career decision-making self-efficacy may raise concern over employment outcomes by strengthening vocational goal engagement, which creates additional psychological pressure. Overall, the associations of parental psychological control and career decision-making self-efficacy on employment anxiety are not unidimensional; they operate through both direct pathways and motivational regulation processes.

Specifically, although parental psychological control exerts a direct positive statistical association with employment anxiety, it produces a negative indirect statistical effect by lowering external regulation and identified regulation. Psychologically controlling family environments are not only linked to heightened subjective stress but also to reduced psychological investment in vocational goals. When people pay less attention to career objectives and employment outcomes, anxiety originating from goal-related pressure may decline accordingly. However, this state of low anxiety does not necessarily correspond to superior vocational adjustment and may merely reflect low vocational engagement. Conversely, career decision-making self-efficacy is directly associated with lower employment anxiety yet generates a positive indirect statistical effect by enhancing identified regulation and external regulation. This implies that high career decision-making self-efficacy is associated with stronger confidence in coping with employment challenges, while simultaneously encouraging individuals to establish clearer vocational goals and intensify their attention to career outcomes. Hence, active vocational engagement and employment-related stress can coexist.

In summary, suppression effects uncover the dual nature of job-seeking motivation. Motivation can be linked to anxiety through intense goal focus, whereas insufficient motivation may temporarily alleviate anxiety. Anxiety levels cannot be judged as simply favorable or unfavorable in isolation; comprehensive evaluation incorporating antecedent environmental conditions and individual psychological resources is required.

## 6 Theoretical contributions and practical implications

### 6.1 Theoretical contributions

Based on Self-Determination Theory (SDT), this study developed a model examining the relationships among parental psychological control, career decision-making self-efficacy, motivational regulation, and employment anxiety. It further revealed the statistical pathways linking environmental factors, individual psychological resources, and motivational mechanisms and employment anxiety among college students. The findings not only provide empirical support for and extend existing theoretical perspectives but also offer new insights into understanding college students’ psychological adaptation in employment contexts.

First, this study extends the application of Self-Determination Theory to the research field of employment anxiety. Previous SDT-based research has primarily focused on the positive effects of autonomy-supportive environments, basic psychological need satisfaction, and motivational internalization on individuals’ development, while paying relatively less attention to the role of motivational regulation processes in high-pressure and uncertain career contexts. By linking parental psychological control, as a controlling social environmental factor, with college students’ employment anxiety, this study reveals how family environments may influence employment anxiety through motivational regulation processes, thereby expanding the application of SDT in explaining individuals’ career-related psychological adaptation.

Second, this study advances the understanding of the relationship between motivational internalization and psychological adaptation within SDT. Traditional SDT emphasizes that higher-level internalized motivation, such as identified regulation, generally facilitates individuals’ positive adaptation. However, this study found that, in the employment context characterized by clear outcome demands and high uncertainty, both identified regulation and external regulation may increase employment anxiety. This finding suggests that motivational internalization does not necessarily lead to positive psychological outcomes, and that its effects may depend on specific situational characteristics and goal-related pressures. Therefore, this study enriches the contextualized understanding of the relationship between motivational quality and psychological outcomes within SDT.

Third, this study contributes to the integration of SDT and career development theories. Previous research on career decision-making self-efficacy has mainly focused on career exploration, career choice behaviors, and career development outcomes. The present study further demonstrates that career decision-making self-efficacy is not only directly associated with employment anxiety but also shows indirect statistical associations with employment anxiety through motivational regulation processes. This indicates that individuals’ career-related competence beliefs influence not only their career behaviors but also the formation of career goals and emotional experiences, providing an integrated theoretical framework for understanding college students’ employment anxiety.

In summary, this study does not simply examine the effects of family factors and self-efficacy on employment anxiety; rather, it reveals the complex role of motivational regulation in employment contexts based on SDT. The findings extend the contextualized interpretation of the relationship between motivational internalization and psychological adaptation within SDT.

### 6.2 Practical implications

This study demonstrates that employment anxiety is statistically associated with family environment, individual psychological resources, and motivational regulation. Therefore, interventions targeting employment anxiety should not be limited to providing employment information or improving job-search skills but should provide multidimensional support based on different influencing pathways.

First, universities should optimize career education and enhance students’ career decision-making abilities. Career guidance programs should shift from focusing solely on employment outcomes to emphasizing students’ overall career development processes. On the one hand, universities can improve students’ abilities in information gathering, career choice, and career planning through systematic career planning courses, career exploration activities, and career decision-making training, thereby enhancing career decision-making self-efficacy. On the other hand, for students with clear career goals but high levels of employment anxiety, universities should not simply strengthen their awareness of employment competition. Instead, they should help students adjust career expectations, encourage them to focus on competence development and long-term growth, and reduce psychological pressure caused by excessive attention to employment outcomes.

Second, families should reduce controlling interventions and support children’s autonomous career choices. Parental psychological control is an important risk factor influencing college students’ employment anxiety. Therefore, family members should avoid interfering with students’ career choices through denial, comparison, or emotional pressure during career development. Instead, they should adopt more supportive communication approaches. For example, parents can participate in students’ career development by providing career information, sharing experiences, and discussing future plans, while respecting students’ own interests, abilities, and career values, thereby facilitating the development of autonomous career goals.

Third, teachers and career counselors should pay attention to the quality of students’ career-related motivation rather than simply increasing their employment motivation. University counselors and career guidance professionals should consider the specific content of students’ employment motivation when providing career support. For students who lack clear career goals, guidance should focus on career value exploration and the development of meaningful career goals. For students who are highly concerned with income, stability, or career achievement, it is necessary to help them establish more diversified career value systems and avoid equating employment success solely with economic returns or occupational stability. In addition, for students with strong career goal commitment but high anxiety levels, interventions should focus on stress regulation and management of outcome expectations rather than merely enhancing employment motivation.

Finally, college students themselves should recognize that employment anxiety does not necessarily originate entirely from insufficient abilities but may also result from excessive attention to career outcomes. During career planning, students should gradually establish career goals based on personal values and competence development, while enhancing psychological resilience in coping with employment uncertainty.

## 7 Conclusion

Based on Self-Determination Theory (SDT), this study examined the statistical associations among parental psychological control, career decision-making self-efficacy, motivational regulation, and employment anxiety among college students. The main findings are as follows: (1) Parental psychological control was significantly and positively associated with college students’ employment anxiety, whereas career decision-making self-efficacy was significantly and negatively associated with employment anxiety. (2) Both parental psychological control and career decision-making self-efficacy were significantly associated with motivational regulation. Specifically, parental psychological control was associated with lower students’ levels of identified regulation and external regulation, whereas career decision-making self-efficacy was linked to facilitated development of both types of motivational regulation. (3) Identified regulation and external regulation significantly mediated the statistical associations between parental psychological control, career decision-making self-efficacy, and employment anxiety, and both mediation pathways showed masking patterns. These two masking pathways may reflect different psychological processes. (4) The associations of different types of career-related motivational regulation with employment anxiety are not unidirectional but may vary depending on employment contexts and individual psychological processes.

### 7.1 Limitations and future directions

Although this study revealed the mechanisms underlying the relationships among parental psychological control, career decision-making self-efficacy, and college students’ employment anxiety, several limitations should be acknowledged.

First, this study employed a cross-sectional questionnaire design. Although structural equation modeling and the Bootstrap method were used to examine relationships among variables, causal directions cannot be determined. Future research could adopt longitudinal designs or experimental approaches to further investigate the dynamic relationships among parental psychological control, changes in career-related motivation, and the development of employment anxiety.

Second, this study focused primarily on parental psychological control and career decision-making self-efficacy as antecedent variables and did not fully consider other important factors in employment contexts. Future studies could incorporate additional variables, such as perceived employment pressure, career adaptability, and social support, to establish a more comprehensive model of employment anxiety.

Third, this study examined the roles of identified regulation and external regulation based on SDT. However, the effects of motivational regulation on psychological outcomes may differ across career development stages and employment environments. Future research could further compare motivational mechanisms among college students with different academic years, majors, or employment stages to deepen the contextualized understanding of the development of employment anxiety.

Fourth, the external regulation subscale showed a Cronbach’s α coefficient of 0.671, which is slightly below the conventional 0.70 threshold. Although its composite reliability (CR = 0.769) meets the acceptable criterion and the 3-item structure often results in lower α estimates, the relatively low internal consistency should be acknowledged. Results related to external regulation should be interpreted with caution, and future research could further optimize the measurement of this construct in employment contexts.

Fifth, situational factors such as perceived employment pressure and outcome uncertainty were not directly measured in this study. Therefore, our contextual interpretation regarding why motivational regulation positively predicts employment anxiety remains a theoretical inference rather than empirically verified conclusion. Future research should incorporate situational moderator measures to test these boundary conditions.

## Data Availability

The original contributions presented in the study are included in the article/supplementary material, further inquiries can be directed to the corresponding author.
